# Smoking cessation reduces systemic inflammation and circulating endothelin-1

**DOI:** 10.1038/s41598-021-03476-5

**Published:** 2021-12-16

**Authors:** Cassandra C. Derella, Martha S. Tingen, Anson Blanks, Samantha J. Sojourner, Matthew A. Tucker, Jeffrey Thomas, Ryan A. Harris

**Affiliations:** 1 Department of Physiology, Augusta University, Augusta, GA USA; 2 Department of Medicine, Georgia Prevention Institute, Augusta University, Augusta, GA USA; 3 Georgia Cancer Center, Medical College of Georgia, Augusta University, 1410 Laney Walker Blvd., CN-2120, Augusta, GA 30912 USA

**Keywords:** Cardiovascular diseases, Physiology

## Abstract

Smoking increases systemic inflammation and circulating endothelin-1 (ET-1), both of which contribute to an elevated risk of cardiovascular disease (CVD). The present study sought to test the hypothesis that a 12-week smoking cessation intervention would contribute to a long-term reduction in circulating ET-1, tumor necrosis factor-alpha (TNF-α), and interleukin-6 (IL-6). 30 individuals participated in a 12-week evidence-based smoking cessation program at Augusta University. Serum cotinine, plasma inflammatory cytokines, and plasma ET-1 were determined at baseline, immediately after the 12-week cessation program (end of treatment, EOT), and 12-months (12M) following the cessation program. Serum cotinine was significantly reduced (*p* < 0.001) at EOT and 12M following the smoking cessation program. Compared to BL (7.0 ± 1.6 pg/mL), TNF-α was significantly reduced at EOT (6.3 ± 1.5 pg/mL, *p* = 0.001) and 12M (5.2 ± 2.7 pg/mL, *p* < 0.001). ET-1 was significantly lower at EOT (1.9 ± 0.6 pg/mL, *p* = 0.013) and at 12M (2.0 ± 0.8 pg/mL, *p* = 0.091) following smoking cessation compared with BL (2.3 ± 0.6 pg/mL). BL concentrations of cotinine were significantly associated with basal ET-1 (r = 0.449, *p* = 0.013) and the change in cotinine at 12M following smoking cessation was significantly associated with the change in plasma ET-1 at 12M (r = 0.457, *p* = 0.011). Findings from the present pilot investigation demonstrate that a 12-week smoking cessation program reduces circulating concentrations of ET-1 and TNF-α for at least a year. The reduction in serum cotinine was associated with the decrease in circulating ET-1. The attenuation in ET-1 and inflammation may in part, contribute to the lower risk of CVD that is observed with smoking cessation.

## Introduction

Nearly 500,000 smoking-related deaths occur each year, resulting in tobacco use as the leading preventable cause of death in the United States^1^. Smoking increases inflammation^2^ and oxidative stress^3^, both of which directly contribute to vascular endothelial dysfunction and subsequent increase in atherosclerosis and cardiovascular disease (CVD) risk^4, 5^. In fact, current smokers are at four times greater risk for developing CVD compared to those whom have never smoked or even those whom have recently quit^6^.

The American Heart Association has identified smoking status (i.e. smoking, never smoked, or quit smoking) as a major component of overall cardiovascular health^7^. If implemented early on, smoking cessation not only reduces excess health hazards by 90%, it can increase life expectancy by up to 10 years^6^. Regardless of smoking history, smoking cessation can favorably impact traditional CVD risk factors, including lowering blood pressure^8^ and reducing systemic inflammation^5^. The endothelin system plays a critical role in regulating vascular endothelial function and both smoking and secondhand smoke can transiently increase circulating concentrations of ET-1^9^ and directly increase the expression of the vasoconstricting endothelin-A receptor^10^. On the other hand, cigarette smoke has multiple deleterious effects on the immune system that can cause immune deficiencies and augment the production of numerous pro-inflammatory cytokines^11^. Interleukin-6 (IL-6) and tumor necrosis factor-α (TNF-α) are arguably two of the most commonly studied pro-inflammatory cytokines^12, 13^, each of which has been reported to increase in both the lungs and circulation with smoking^2, 14^.

Although 70% of smokers express interest in quitting, most are discouraged by the high failure rates and only ~ 8% of smokers are successful at quitting without guidance^1^. Interestingly, the success of smoking cessation is more closely associated with the degree of nicotine dependence than the motivation to quit^15^. The success of a smoking cessation program is also directly related to the type of intervention (group or individual therapy), the number of intervention modalities, and the number of reinforcing sessions. Therefore, inclusion of the evidence-based components of both pharmacotherapy (i.e., to address the physical addiction) and behavioral skills counseling (i.e., to address the psychological addiction) are essential for smoking cessation success^16, 17^.

Smoke exposure increases ET-1^18^ and augments systemic inflammation^19^; however, the effect of a short-term (12-week) smoking cessation program on ET-1 and markers of inflammation over time is unknown. Accordingly, the present pilot investigation sought to test the hypothesis that a 12-week smoking cessation program would contribute to a reduction in ET-1, TNF-α, and IL-6 over a 12-month post cessation period.

## Methods

### Experimental design

**Figure 1:**
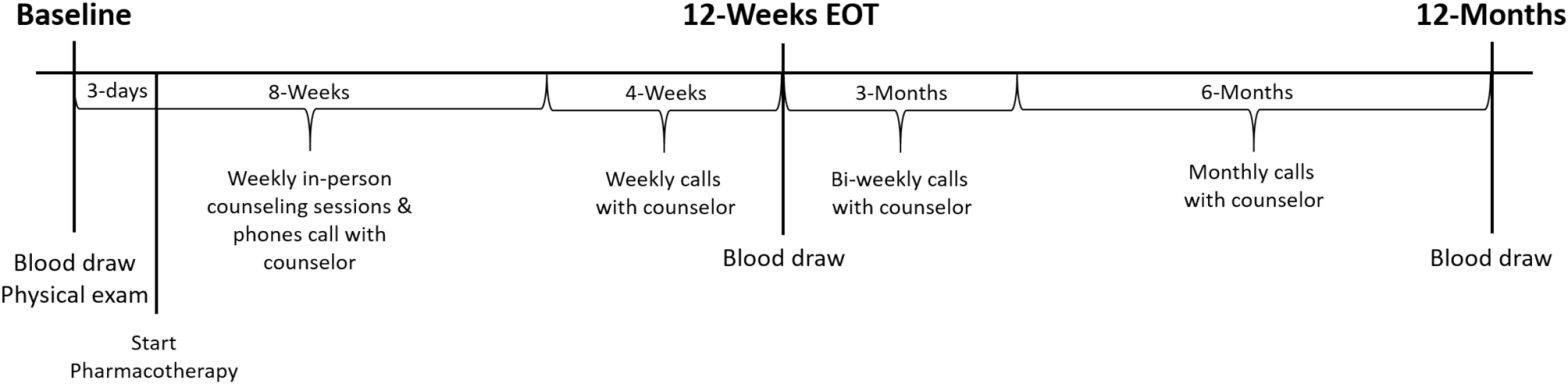
Timeline of the smoking cessation program and follow-up visits.

The present investigation was comprised of three experimental visits: baseline (BL), 12-weeks (end of treatment, EOT), and 12-months (12M). The study timeline is presented in Fig. 1.

The three experimental visits occurred at various times throughout the day to accommodate participant’s schedules. The BL visit included an initial physical exam by a board certified nurse practitioner (NP) who was able to help assess the best pharmacotherapy in discussion with the participant. On each follow-up visit (EOT and 12M), demographics and vitals were collected and a venous blood sample was obtained. The NP was available at these two visits and seen by the participant only if there were concerns with the vital signs or any problems with the prescribed smoking cessation pharmacotherapy.

### Participants

Thirty-five volunteers agreed to participate in this study. Participants were eligible if they met the following criteria: (1) were ≥ 18 years of age, (2) scored at least a 6 out of 10 on the Readiness to Quit Ladder^20^, which indicates “I definitely plan to quit smoking within the next 6 months,” and (3) were either apparently healthy or if with chronic illness(es), were well controlled on a treatment plan. Participants who were scheduled for a cessation clinic appointment and met the inclusion criteria were invited to enroll in the study. Research participants completed the written informed consent document (ICD) process after all questions were answered by the principal investigator or a study team member trained in the ICD process. This study was approved by the Institutional Review Board at Augusta University and all experiments were performed in accordance with relevant guidelines and regulations.

### Smoking cessation program including pharmacotherapy

The smoking cessation program was 12-weeks long. For the first 8-weeks, participants were contacted by phone each week and attended weekly in-person counseling sessions with a cessation counselor/coach. In-person coaching sessions were conducted in groups of 6–10 with each session lasting 45–60 min. Topics that were addressed and covered during these sessions included the following: the effects of nicotine on the body, physical and psychological addiction of smoking, triggers to smoking, and the importance of being prepared when tempted and with cravings. The format was an open discussion with a cessation counselor who was experienced in both tobacco treatment and motivational interviewing, a technique shown to be effective with smoking cessation^16, 17^. Following the conclusion of the 12-week smoking cessation program, participants were contacted to monitor smoking cessation status every 2 weeks for 3-months and then monthly for the remaining 6-month period.

Participants started their respective pharmacotherapy within 3-days of the baseline visit. Each participant was prescribed an individual pharmacotherapy based on the recommendation of the NP on the initial visit. The pharmacotherapies included a daily oral dose of Varenicline (1 mg/day for the first week, then increased to 2 mg/day) or use of a nicotine replacement therapy (NRT), specifically the patch. Initial pharmacotherapy was prescribed for a month at the time and participants were instructed to take the medication minimally for 8 weeks and at maximum for 16-weeks. Any side effects experienced from the pharmacotherapy were reported to the counselor who contacted the NP immediately to address as needed. Compliance of the pharmacotherapy was measured at each experimental visit by self-report. Participants were asked if they were still using the therapy, if so, how often and if not, when they last used the therapy. This study was completed prior to any of the current concerns and holds on varenicline usage.

### Blood analysis: cotinine, ET-1, TNF-a and IL-6

A venous blood sample was collected into serum separator or EDTA treated collection tubes (BD, United States) and serum or plasma, respectively, was separated out by centrifugation. Serum and plasma samples were flash frozen in liquid nitrogen and stored at − 80 °C until later analyzed.

Cotinine, the primary metabolite of nicotine^21^, is considered the “gold standard” for the assessment of smoking status, including verification of smoking cessation^22^. Serum cotinine was assessed at BL, EOT, and 12M using an established protocol of liquid chromatography and mass spectrometry by Salimetrics, LLC. Concentrations of plasma ET-1, TNF-α, and IL-6 were determined using a Simple Plex cartridge on the Ella Platform (Protein Simple, San Jose, CA) according to the manufacturer’s instructions. The limit of detection of human ET-1, TNF-α, and IL-6 are 0.250–1000 pg/mL, 1.14–290 pg/mL, and 0.41–3850 pg/mL, respectively.

### Statistical analyses

All statistical analyses were performed using SPSS version 24 (SPSS Inc, Chicago, IL). All data are presented as mean ± standard error of the mean unless otherwise noted. All data was assessed for normal distribution and sphericity. Mauchly’s test of sphericity was performed and if rejected, the Greenhouse–Geisser correction was applied. A repeated measures analysis of variance (ANOVA) was performed to determine changes in participant characteristics, serum cotinine, and plasma ET-1, IL-6, and TNF-α over the 12-month study period. When a significant main-effect was found, Tukey’s post-hoc analysis with Bonferroni correction was used. Pearson’s correlations were performed to evaluate the relationship between serum cotinine and circulating ET-1. Statistical significance was set a priori to an alpha level of ≤ 0.05.

### Ethics approval and consent to participate

This study was approved by the Institutional Review Board at Augusta University (IRB Number: 0805252). Research participants completed the written informed consent document (ICD) process prior to participation, after all questions were answered by the principal investigator or a study team member trained in the ICD process.

## Results

### Participant characteristics

During the enrollment period, 35 participants agreed to participate in the pilot study. Of these 35 individuals, 31 (89%) completed all three experimental visits, and the cessation counseling sessions. Four participants (11%) were lost to follow-up: 2 chose to stop participating in the study prior to its conclusion, and 2 were non-responsive to all follow-up attempts to reach them. For the purpose of this study, one participant was excluded from the final analysis due to insufficient plasma for the ET-1 and inflammatory marker analysis. Participant characteristics are presented in Table 1.

**Table 1:** Participant characteristics.

Variable	Baseline	EOT	12-Month
N	30	–	–
Men	15	–	–
Women	15	–	–
Race (%)			
Caucasian	71	–	–
African American	20	–	–
Asian	6	–	–
Hispanic	3	–	–
Age (years)	37 ± 11	37 ± 11	38 ± 10
Height (cm)	169.5 ± 2.0	169.9 ± 2.0	170.8 ± 2.0
Weight (kg)	80.0 ± 3.4	81.5 ± 3.7	83.9 ± 3.6*^†^
BMI (kg/m^2^)	27.8 ± 1.0	28.1 ± 1.1	28.6 ± 1.1
SBP (mm Hg)	130 ± 3	129 ± 2	130 ± 2
DBP (mm Hg)	83 ± 1	81 ± 2	80 ± 2
MAP (mm Hg)	99 ± 2	97 ± 1	97 ± 2

Participant characteristics analyzed for changes between visits.

Significant values are in bold.

*EOT* end of treatment (3-months), *BMI* body mass index, *SBP* systolic blood pressure, *DBP* diastolic blood pressure, *MAP* mean arterial pressure.

*Indicates significant difference from Baseline.

^†^Indicates significant difference from EOT; Repeated measures ANOVA.

Compared to BL, body weight was significantly (*p* = 0.001) higher at 12M. There was no change in any other anthropometric variables during the 12-week smoking cessation program or throughout the 12M study period. The pharmacotherapies, treatment compliance and the side-effects experienced while on the therapy are presented in Table 2.

**Table 2:** Pharmacotherapies, compliance and side-effects.

Variable	Baseline	EOT	12-Month
**Pharmacotherapies**			
Varenicline	94%	33%	10%
Patch	3%	3%	3%
None	3%	64%	87%
**Self-reported smoking status**			
Still smoking	100%	67%	53%
No longer smoking	0%	33%	47%
**Common side-effects**			
*Varenicline*	n = 28	n = 9	n = 3
Vivid dreams	–	50%	39%
Nausea	–	29%	18%
GI issues (flatulence, cramps, aches, etc.)	–	14%	4%
Loss of appetite	–	4%	–
Insomnia	–	11%	–
Constipation	–	4%	7%
Irritability/mood swings	–	4%	4%
Inability to focus	–	4%	–
Fatigue	–	4%	–
Depression	–	–	4%
None	–	21%	14%
*NRT-patch*	n = 1		
None	–	100%	100%

The pharmacotherapies prescribed and the percentage of participants on each at each experimental visit. Self-reported smoking status was recorded at each experimental visit and side-effects from the pharmacotherapies experienced throughout the study. Participants may have restarted pharmacotherapy to help relapses and urges.

Twenty-eight participants were prescribed an oral dose of Varenicline, one participant used NRT (patch), while one did not use any pharmacotherapy. Although pharmacotherapies were prescribed for a maximum of 16 weeks, participants could stop treatment prior to EOT if successful with quitting and restart the pharmacotherapy after EOT to help resist urges and/or if they had relapsed.

### Biochemical analyses

Concentrations of serum cotinine at BL, EOT, and 12M are presented in Table 3. Compared to BL, a significant decrease in cotinine was observed at EOT (*p* < 0.001) and persisted through the 12M visit (*p* < 0.001).

**Table 3:** Biochemical analysis of ET-1, cotinine, Il-6, and TNF-α.

Variable	Baseline	EOT	12-Month
Cotinine (ng/mL)	176.8 ± 109.5	45.0 ± 89.9*	74.9 ± 94.3*
ET-1 (pg/mL)	2.3 ± 0.6	1.9 ± 0.6*	2.0 ± 0.8
TNF-α (pg/mL)	7.0 ± 1.6	6.3 ± 1.5*	5.2 ± 2.7*^†^
IL-6 (pg/mL)	2.19 ± 1.09	1.84 ± 0.75	1.80 ± 1.13

Serum concentrations of cotinine (n = 30) and plasma concentrations of ET-1 (n = 30), TNF-α (n = 30), and IL-6 (n = 28) at baseline, 3-months (end of treatment, EOT), and 12-months (12M) following smoking cessation. Repeated measures ANOVA.

*p < 0.05 compared with BL.

^†^*p* < 0.05 with 3M.

Concentrations of circulating ET-1, TNF-α, and IL-6 at BL, EOT, and 12M are presented in Table 3. Compared to BL, concentrations of ET-1 were significantly reduced at EOT (*p* = 0.013) and this reduction was maintained at 12M (*p* = 0.091). Compared to BL, TNF-α was significantly (*p* = 0.001) lower at EOT. In addition, circulating concentrations of TNF-α continued to decrease out to 12M compared to both BL (*p* < 0.001) and EOT (*p* = 0.002). IL-6 data for all three time points were only available for 28/30 participants due to insufficient sample volume at 12M for two participants. Although not statistically significant, IL-6 tended (*p* = 0.114) to decrease from BL at EOT and the decrease was sustained at 12M.

**Figure 2:**
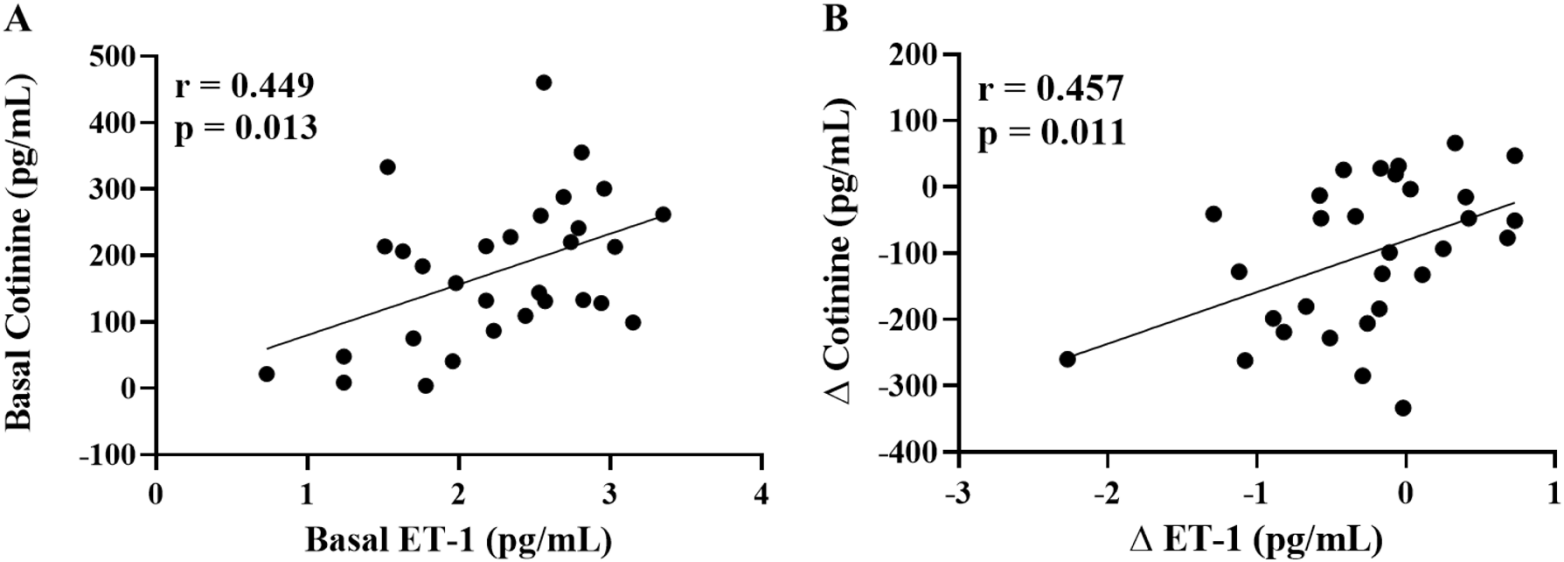
Relationship between basal values of cotinine (n = 30) and ET-1 (**A**) and the change in cotinine and ET-1 following 12-months of smoking cessation (**B**). Pearson’s correlation (r). Δ is the change in cotinine or ET-1 from baseline to 12-month.

Figure 2A illustrates the significant correlation between concentrations of cotinine and circulating ET-1 (r = 0.449, *p* = 0.013). In addition, the change in cotinine at 12M following smoking cessation was positively associated with the change in ET-1 at 12M (r = 0.457, *p* = 0.011) (Fig. 2B).

## Discussion

Smoking can have a negative impact on circulating concentrations of ET-1^9^ and systemic inflammation^19^, both of which are independent risk factors of CVD. Smoking cessation can significantly reduce the overall risk of CVD^6^; however, whether or not smoking cessation is associated with changes in ET-1 and inflammatory status had not as yet been determined. The present investigation sought to test the hypothesis that a 12-week, evidence-based smoking cessation intervention would contribute to a long-term reduction in circulating concentrations of ET-1, TNF-α, and IL-6. Findings of the present investigation demonstrate that a 12-week smoking cessation intervention is effective at not only reducing both circulating ET-1 and TNF-α, but also sustaining the reduction for at least 12-months. Perhaps most importantly, the reduction in circulating cotinine over time was associated with the reduction in plasma ET-1.

### Efficacy of the smoking cessation program

A ‘former smoker’ is defined by the US Centers for Disease Control and Prevention as a non-smoking adult that has smoked at least 100 cigarettes in their lifetime^23^. It often takes several attempts for a smoker to successfully quit smoking and self-reported measures of smoking and smoking cessation are highly imprecise and inaccurate^21^. Cotinine, the major metabolite of nicotine, is regarded as the most objective biochemical measure of smoke exposure^22^. The concentration of cotinine is positively related to the amount of nicotine an individual is exposed to, with greater exposure eliciting higher concentrations of cotinine^21^. When an individual quits smoking, concentrations of cotinine begin to decline, and with prolonged cessation, cotinine concentrations decrease to undetectable levels^21^. The significant reduction in serum cotinine after 12-weeks of smoking cessation in the present investigation confirms the adherence and effectiveness of our evidence-based program. The average serum concentrations of cotinine at EOT was 47.0 ng/mL, indicative of a reduction in smoking but not 100% adherence. These results suggest a reduction in smoking frequency; however, this was not a primary focus of the present study. Importantly, compared to BL, serum cotinine remained lower at 12M and indicates that many participants continued to abstain from smoking for at least 9-months after the 12-week cessation program had ended.

### Smoking cessation and endothelin-1

ET-1 is a potent vasoconstrictor peptide that has an important pathological role in the pathogenesis of hypertension^24, 25^ and overall CVD risk^26^. ET-1 provides opposing actions by acting through a vaso-constricting ETA-receptor (ET_A_R) and a vaso-dilating ETB-receptor (ET_B_R)^27^. Additionally, ET-1 increases renal vascular constriction and proteinuria in the kidney^26^, all of which contribute to the development of hypertension and subsequent CVD. The baseline concentrations of ET-1 measured in this study are consistent with previous investigations that have documented an increase in circulating concentrations of ET-1 with active smoking^9^. Findings of the present investigation are the first to demonstrate that (1) a short-term smoking cessation program elicits a significant reduction in circulating ET-1, and (2) the reduction in ET-1 is maintained for at least 12M with continued cessation. Perhaps most relevant, those who exhibited the greatest reductions in cotinine at 12M had the greatest reductions in circulating ET-1. The reduction in circulating ET-1 limits the number of ligands available to bind to the potent ET_A_R, thereby attenuating acute vasoconstriction and improving vascular tone^25, 27^. Taken together, these data suggest that the decline in cotinine with smoking cessation may contribute in part, to the decrease in circulating ET-1. Nonetheless, the evidence-based smoking cessation program at Augusta University resulted in a significant decrease in circulating ET-1, which likely contributes to an overall reduction in CVD risk.

### Smoking and markers of inflammation

Chronic inflammation also plays a central role in the development of CVD and a reduction in inflammation offers cardio-protection^28^. In particular, there is great interest in targeting a decrease in TNF-α^28^ and IL-6^29^, two hallmark pro-inflammatory cytokines. Elevated inflammatory cytokines have a direct impact on vascular resistance and endothelial cell apoptosis and both TNF-α and IL-6 can be stimulated by smoking^19^. As a vicious cycle, smoke exposure results in immediate tissue damage and this damage elicits an inflammatory immune response^2^ that continues to aid in repair. Chronic inflammation, such as that observed with long-term smoking, is associated with CVD^14^. Consistent with previous reports^30^, baseline concentrations of inflammation were elevated in the participants of the present investigation. With smoking cessation; however, a significant reduction in TNF-α was observed at EOT and persisted lower at 12M. In addition, IL-6 was reduced following the 12-week smoking cessation program, although it did not reach statistical significance. Previous studies have suggested that statistically significant reductions in IL-6 do not occur until 5–9 years following smoking cessation^31, 32^. Nonetheless, the reductions in circulating IL-6 and TNF-α observed in the present investigation may be clinically significant as the decrease in each individual cytokine may act synergistically to contribute to an overall greater reduction in CVD risk. In fact, targeting risk factors of CVD with a multifactorial approach has been proposed to be more effective in lowering CVD risk compared with modifying a single risk factor by itself^33^. Taken together, findings of the present investigation provide evidence that a short-term smoking cessation program results in an overall reduction in the systemic inflammatory profile.

### Experimental considerations

In addition to elevated ET-1 at baseline, participants of the current investigation were classified as having stage 1 hypertension^34^, with an average blood pressure of 130/83 mm Hg. While not statistically significant, there was a modest, but clinically meaningful reduction in mean arterial pressure (MAP; 2 mm Hg) following cessation that may also have contributed to a reduction in CVD risk^35^. Interestingly, smoking cessation has been observed to coincide with an increase in blood pressure, particularly in those who had quit for over a year^36^. The modest change in blood pressure we observed in the current investigation could be due to (1) a less than 100% adherence in quitting and/or (2) we only tracked our participants out for 1 year. Reductions in ET-1 are associated with reductions in blood pressure^37^, which could, in part, explain the clinically significant reduction in MAP.

It is important to note that sex and race differences have been identified throughout the endothelin system^38, 39^. Although an equal number of men and women were recruited for this study, no sex differences in any outcomes were identified. In addition, the present study was not powered to test the effects of race as only 20% of our cohort identified as African Americans. In a similar line, this pilot study only included a small sample size of 30 individuals. Future studies are warranted to replicate the results of the current study in a larger cohort considering equal representation of sex and race.

Vascular dysfunction is not only a consequence of a heightened endothelin system^27^ but smoking as well^40^. While outside the scope of the current investigation, inclusion of other vascular measures, such as flow-mediated dilation, arterial stiffness or microvascular function could provide further insight into the CVD risk benefits of reducing ET-1 with smoking cessation. In a similar line, changes in nutrition^41^, alcohol intake^42^, or exercise^43^ have a strong interrelationship with smoking and smoking cessation. Smoking has appetite suppressive effects that often leads to weight loss^44^. Therefore, it is not surprising that we observed a significant increase in weight throughout the study. It is important to note; however, that we cannot rule out the impact of dietary or exercise changes that may have occurred during the treatment period, which can affect body weight. While not measured in the current study, future investigations that incorporate lifestyle and behavior and vascular health assessments with biomarkers are warranted.

In addition, further research is needed that examines the relationship between nicotine dependence of smokers [typically measured using the Fagerstrom Test for Nicotine Dependence (FTND)] and both ET-1 and inflammatory markers. Findings may provide important information on possible linkages between dependence and inflammatory markers pre and post-cessation as well as provide insight into the impact of the addiction level on the success of quitting. Although the pharmacotherapy prescribed was based on the number of cigarettes smoked per day, which is an acceptable measure of nicotine addiction and dependence, this study used the Readiness to Quit Ladder as inclusion criteria versus the FTND. Nonetheless, the FTND is an important test for addiction and future studies should include this factor due to the novel and important findings that may emerge.

## Conclusions

The positive effects of smoking cessation on overall cardiovascular health are well established^5^. However, there are numerous factors that can contribute to the increased CVD risk with smoking, such as elevated ET-1^26^ and inflammation^19^. Findings from the present investigation demonstrate that an evidence-based 12-week smoking cessation program is not only effective, it can also reduce circulating concentrations of ET-1, TNF-α, and IL-6, all of which can contribute to long-term reductions in CVD risk. Perhaps most important, the change in cotinine was associated with the change in ET-1, suggesting that reductions in CVD risk associated with smoking cessation may in part, be due to the decrease in circulating ET-1.

## Data Availability

The datasets used and/or analyzed during the current study are available from the corresponding author on reasonable request.

## Abbreviations

ET-1: Endothelin-1
CVD: Cardiovascular disease
TNF-α: Tumor necrosis factor-alpha
IL-6: Interleukin-6
BL: Baseline
EOT: End of treatment
12M: 12-Months
NP: Nurse practitioner
ICD: Informed consent document
NRT: Nicotine replacement therapy
ANOVA: Analysis of variance
BMI: Body mass index;
SBP: Systolic blood pressure
DBP: Diastolic blood pressure
MAP: Mean arterial pressure
ET_A_R: ETA-receptor
ET_B_R: ETB-receptor
